# Recent Advances in Improving the Alkaline Oxygen Reduction Performance of Atomically Dispersed Metal–Nitrogen–Carbon Catalysts

**DOI:** 10.3390/nano15161257

**Published:** 2025-08-15

**Authors:** Jian Chen, Zheng Li, Xiong Du, Mengran Wang, Simin Li, Qiyu Wang, Yangen Zhou, Yanqing Lai

**Affiliations:** 1 School of Metallurgy and Environment, National Energy Metal Resources and New Materials Key Laboratory, Hunan Provincial Key Laboratory of Nonferrous Value-Added Metallurgy, Central South University, Changsha 410083, China; 213507010@csu.edu.cn (J.C.); duxiongcsu@csu.edu.cn (X.D.); 222034@csu.edu.cn (M.W.); simin.li@csu.edu.cn (S.L.); laiyanqing@csu.edu.cn (Y.L.); 2 School of Materials Science and Engineering, Hunan University of Science and Technology, Xiangtan 411201, China; lizheng@hnust.edu.cn; 3 Research Institute of Renewable Energy and Advanced Materials, Zijin Mining Group Co., Ltd., Changsha 410013, China

**Keywords:** metal–air batteries, oxygen reduction reaction, M-N-C catalyst, single-atom catalyst

## Abstract

Atomically dispersed metal–nitrogen–carbon (M-N-C) catalysts are regarded as ideal catalytic materials for the oxygen reduction reaction (ORR) under alkaline conditions. Compared with other ORR catalysts, M-N-C catalysts exhibit notable advantages, including low cost, high atomic utilization efficiency, and considerable catalytic potential. We provide a systematic review of recent research advances in enhancing the ORR performance of M-N-C catalysts, focusing on catalytic activity and stability. First, the reaction mechanism of the ORR on the surfaces of the M-N-C catalysts is elucidated. Second, the primary strategies employed in recent years to improve their catalytic activity and stability are summarized. Finally, critical research directions that should be prioritized to expedite the commercialization of M-N-C catalysts are outlined.

## 1. Introduction

To achieve the “dual carbon” goals, the development and application of clean and efficient green new energy sources have become focal areas of research and practice. As a promising energy conversion technology, metal–air batteries are considered a leading solution to address the growing global energy crisis and environmental issues. This is attributed to their high theoretical energy density, straightforward design, and benefits in terms of safety and eco-friendliness [1,2]. Compared with acidic and neutral electrolytes, which are associated with various limitations, alkaline electrolytes represent a more favorable option for metal–air batteries. However, the efficiency of these batteries is hindered by the slow oxygen reduction reaction (ORR) occurring at the cathode, which has greatly delayed their widespread commercial adoption. Thus, the exploration and development of highly effective alkaline oxygen reduction catalysts are essential to promote the practical application and commercial viability of metal–air batteries.

ORR catalysts can be categorized based on their composition into Pt-based catalysts, transition metal compound catalysts, and carbon-based catalysts. Pt-based materials are considered optimal catalysts for the ORR because they exhibit favorable adsorption energy toward oxygen-containing intermediate species [3]. However, the scarcity and high cost of Pt constrain the practical application of Pt-based catalysts. To balance high catalytic activity with reduced Pt consumption, two primary strategies are currently employed to enhance their catalytic performance. The first strategy involves alloying control [4,5]. Wang et al. aimed to reduce Pt consumption through a synergistic design of composition and structure [6]. The as-synthesized Pd@Pt_2.7_L/C catalyst, obtained by precisely tuning the Pd core structure and the Pt/Pd mass ratio, demonstrates a half-wave potential (E_1/2_) of 0.9 V, accompanied by a kinetic current density that is 7.8-fold greater than that of commercial Pt/C. However, the increasing cost of Pd constrains its application potential. The second strategy involves structural optimization. Chen et al. synthesized hollow Pt_3_Ni nanocages through the etching of PtNi_3_ crystals [7]. The porous structure exposes a greater number of active sites, while the incorporation of Ni optimizes the electronic structure of Pt, leading to an E_1/2_ of 0.91 V and a Tafel slope as low as 46 mV dec^−1^. Wang et al. synthesized an ultra-thin Pt shell with tungsten carbide as the core [8], whereas Tian et al. constructed a Pt shell–titanium nickel nitride core structure (with a Pt content of 5 wt%) [9]. Both strategies significantly improved the ORR catalytic activity through enhanced Pt dispersion and *d*-band center modulation. Although the aforementioned strategies reduce Pt consumption while maintaining high catalytic activity, Pt is prone to oxidation into low-conductivity oxides during the long-term operation of metal–air batteries. Furthermore, its relatively high cost continues to hinder large-scale applications.

Catalysts based on transition metals are considered viable alternatives to Pt-based catalysts, as the e_g_ orbitals of the metal atoms show significant overlap with the 2*p* orbitals of O_2_, which facilitates stronger O-O bond coupling. These catalysts are primarily classified into four categories: oxides, carbides, sulfides, and phosphides. Despite the natural abundance of transition metal oxides, including spinel and perovskite structures, their catalytic performance is frequently constrained by poor electrical conductivity and strong surface hydrophilic characteristics. Through the metal atom transposition effect, the charge transfer efficiency of this type of catalyst can be markedly enhanced, leading to improved ORR activity [10]. Transition metal carbides such as tungsten carbide (WC), formed by the embedding of carbon atoms into metal lattices, possess a Pt-like electronic structure and can demonstrate excellent ORR performance. Guo et al. prepared a tungsten carbide (WC) nanoparticle (NP) catalyst with an average particle diameter of 1.9 nanometers [11]. This structural design enhances electrical conductivity while effectively preventing the aggregation of tungsten carbide nanoparticles, leading to excellent ORR performance and a favorable four-electron reaction pathway. The multivalent characteristics of sulfur and phosphorus elements endow transition metal sulfides and transition metal phosphides with dynamic redox capabilities. Dou et al. synthesized a plasma-treated Co_9_S_8_/GO catalyst exhibiting a half-wave potential of 0.84 V, which is comparable to that of commercial Pt/C [12]. Although considerable advancements have been achieved in enhancing the catalytic performance of transition metal-based catalysts, their catalytic activity toward the ORR remains insufficient to meet the demands of practical applications. Additionally, their poor electrical conductivity and restricted surface area impede both electron transfer and mass transport during the ORR. In addition, such catalysts exhibit weak binding affinities toward O_2_ and oxygen-containing intermediates, along with pronounced surface electric field effects, which increase the difficulty of O-O bond cleavage. Consequently, the ORR catalytic activity remains suboptimal.

Carbon-based catalysts have gained significant attention as potential materials for ORR catalysis owing to their excellent electrical conductivity, extensive surface area, and economic viability. Their catalytic activities originate from the asymmetric charge distribution induced by the incorporation of defects or impurity atoms into the sp^2^-hybridized carbon skeleton, which enhances their capacities to adsorb and chemically interact with O_2_ [13]. Presently, carbon-based catalysts can be broadly divided into three main groups. The first group includes pristine carbon materials such as graphene, carbon nanotubes, and carbon derivatives obtained from metal–organic frameworks (MOFs). The catalytic activity of pure carbon materials can typically be enhanced through defect engineering. Highly defective carbon quantum dots synthesized via an interfacial self-corrosion strategy achieve an E_1/2_ of 0.90 V under alkaline conditions [14]. However, their complex synthesis process hinders practical applications. The second group consists of non-metal-doped carbon materials, with nitrogen-doped carbon materials attracting significant interest owing to their advantageous electronegativity balance and superior electron-donating properties. Nitrogen-doped carbon materials can facilitate O_2_ mass transfer by constructing mesoporous structures, and their catalytic activity can even approach or rival that of Pt-based catalysts [15]. In contrast to the first two categories of carbon materials, transition metal-doped carbon materials possess a greater abundance of active sites for the ORR. As far back as 1964, Jasinski and colleagues identified the catalytic reduction activity of M-N_4_ macrocyclic complexes, where M stands for a transition metal and N represents a nitrogen atom coordinated to the metal [16]. In 2010, studies explored affordable nitrogen-rich precursors, inorganic metal sources, and carbon supports with high surface areas as economical substitutes for costly metal-macrocyclic compounds in the synthesis of M-N-C catalysts, achieving outstanding electrocatalytic activity [17,18,19]. At present, transition metal-doped carbon catalysts, especially atomically dispersed transition metal–nitrogen–carbon (M-N-C) catalysts, demonstrate numerous highly active sites and are considered the most viable contenders for ORR catalytic applications (Table 1).

**Figure 1:**
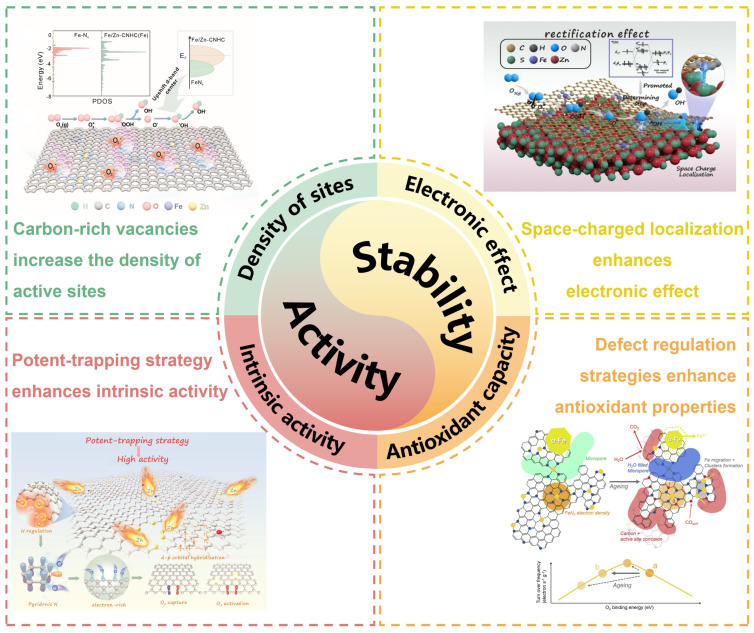
Typical strategies for improving the ORR performance of M-N-C catalysts. Images for “Carbon-rich vacancies increase the density of active sites”: Reproduced with permission [46]. Copyright 2023, Elsevier. Images for “Space-charged localization enhances electronic effect”: Reproduced with permission [47]. Copyright 2025, Elsevier. Images for “Potent-trapping strategy enhances intrinsic activity”: Reproduced with permission [48]. Copyright 2023, Wiley-VCH. Images for “Defect regulation strategies enhance antioxidant properties”: Reproduced with permission [49]. Copyright 2020, Elsevier.

Catalytic activity and stability are the two main criteria for evaluating the ORR performance of M-N-C catalysts. Here, we provide a systematic and comprehensive overview of recent research advances aimed at enhancing the catalytic activity and stability of M-N-C catalysts (Figure 1). Based on current research progress, this paper outlines several strategically forward-looking development directions aimed at accelerating the commercialization of M-N-C catalysts.

## 2. Advances in Research on M-N-C Catalysts

**Figure 2:**
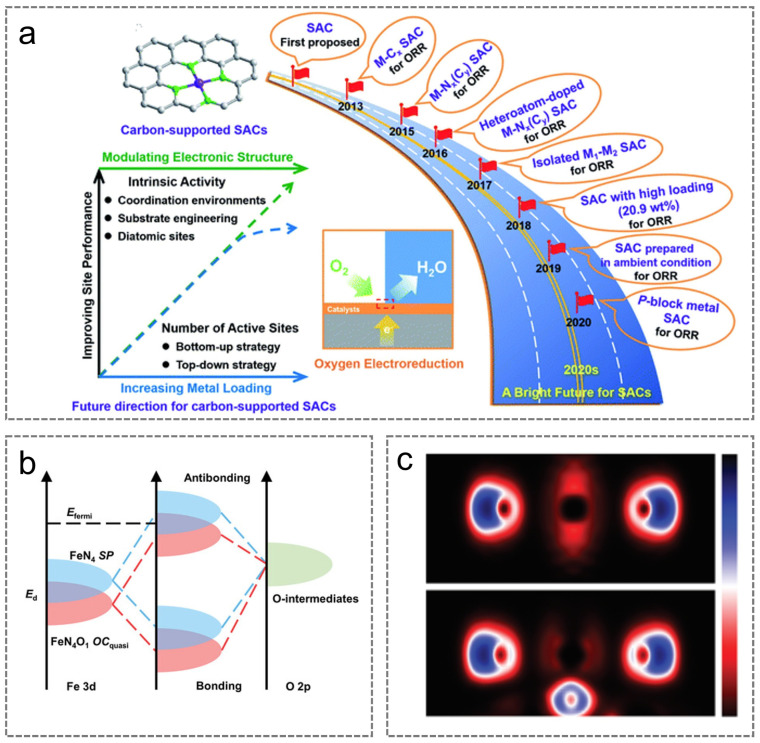
(**a**) Structure image of M-N-C catalysts. Reproduced with permission [50]. Copyright 2021, Wiley-VCH. (**b**) ORR hybridization process image. (**c**) Calculated charge density differences for M-N-C catalysts. (**b**,**c**) Reproduced with permission [55]. Copyright 2023, Wiley-VCH.

M-N-C catalysts feature metal active sites (M-Nx) that are atomically dispersed and anchored onto a carbon-based framework (Figure 2a). However, due to carrier interactions and reaction conditions, the charge state of metal sites is predominantly cationic rather than neutral. The enhanced electronic structure of M-N-C catalysts arises from the coordination interaction between the transition metal, M, and nitrogen atoms as well as their synergistic interaction with the carbon matrix [50]. As illustrated in Figure 2b, the *d*-band of the M atom in the M-N-C catalyst is broader compared with that of pure metal, and the *d*-band center shifts negatively away from the Fermi level. These phenomena, resulting from the hybridization of different *p* orbitals of nitrogen atoms, facilitate the adsorption and desorption of oxygen-containing intermediates. Secondly, the electronegativities of M and N in M-N-C differ, leading to charge transfer from M to N (Figure 2c). As the *d*-band becomes increasingly electron-rich, its hybridization with the oxygen *p* orbitals is enhanced, which facilitates the cleavage of the O-O bond. Ultimately, integrating M-N_x_ sites within the carbon matrix promotes the generation of additional pyrrolic nitrogen rings, thereby enhancing the catalytic reaction rate [51,52,53,54,55].

To confirm the atomic-level dispersion of M-N-C catalysts, a combination of multiple characterization techniques is typically required, such as aberration-corrected transmission electron microscopy (AC-TEM) and X-ray absorption spectroscopy (XAFs). AC-TEM can directly visualize the dispersion of metal atoms, thereby confirming their isolated existence. XAFs can detect the presence of metal–metal bonds in materials, thereby indicating whether the metals are atomically dispersed. Additionally, Mössbauer spectroscopy, X-ray photoelectron spectroscopy (XPS), and electron paramagnetic resonance spectroscopy (EPR) can serve as complementary techniques to further confirm the atomic-level dispersion of metals on supports.

### 2.1. Oxygen Reduction Mechanism and Current Challenges of M-N-C Catalysts

**Figure 3:**
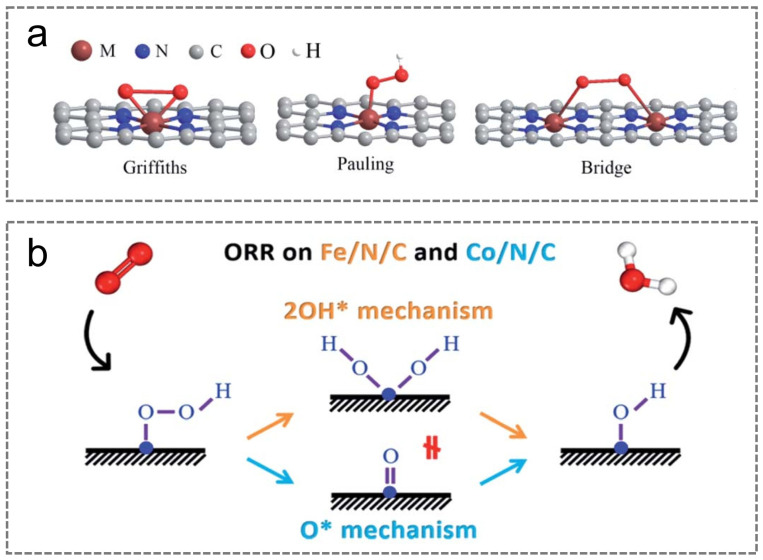
(**a**) Schematic depiction of three distinct oxygen molecule adsorption models. Reproduced with permission [57]. Copyright 2020, Royal Society of Chemistry. (**b**) Diagram depicting the *O pathway and the 2*OH reaction mechanism. Reproduced with permission [58]. Copyright 2020, American Chemical Society.

The ORR reaction pathway of M-N-C catalysts is predominantly determined by the adsorption and subsequent condensation behavior of O_2_ on the catalyst surface [38,56,57]. There are three main types: Griffiths, Pauling, and Bridge (Figure 3a). In the Griffiths type, the strong coupling between the d orbitals of the M atom and oxygen molecules leads to a weakening of the O-O bond, thus guiding the ORR to follow a four-electron reaction mechanism. In the Pauling type, only one oxygen atom within the O_2_ molecule interacts with the M atom, which hinders O-O bond cleavage and results in the ORR proceeding via a two-electron reaction pathway, thereby negatively impacting catalytic activity. The Bridge configuration is applicable to M-N-C catalysts containing two or more adjacent M atoms, where oxygen molecules are simultaneously adsorbed on two metal sites. This structural feature facilitates O-O bond cleavage and allows the ORR to progress through a four-electron reaction route [58].

Additionally, based on whether the O-O bond cleavage occurs before proton–electron transfer, the ORR reaction routes on M-N-C catalysts can be categorized into dissociative and associative pathways (Figure 3b). Both pathways involve key oxygen-containing intermediates (*O and *OH), whereas *OOH is exclusively present in the association pathway. The binding energies of these oxygen-containing intermediate species on the surface of M-N-C catalysts are strongly related to the location of the metal *d*-band center [59]. Typically, the binding strength of oxygen-containing intermediates on catalyst surfaces should not be too strong or too weak. If the binding strength is too high, reaction intermediates may continuously occupy the catalyst’s active surface, thereby impeding the ongoing progress of the catalytic process. Conversely, if the adsorption strength is too weak, it can impede reactant adsorption and subsequent desorption processes [60]. In addition, an unconventional 2*OH mechanism has been proposed: for traditional catalysts featuring similar, uniformly distributed active sites (M-N_x_), 2ΔG(*OH) = ΔG(*O). However, for M-N-C catalysts, ΔG(2*OH) = ΔG(*O) + 1.5 eV; this indicates that the 2*OH reaction pathway should not be overlooked [61]. At the same time, it was observed that the Co-N-C catalyst prefers the *O mechanism, whereas the Fe-N-C catalyst favors the 2*OH reaction pathway [62].

**Figure 4:**
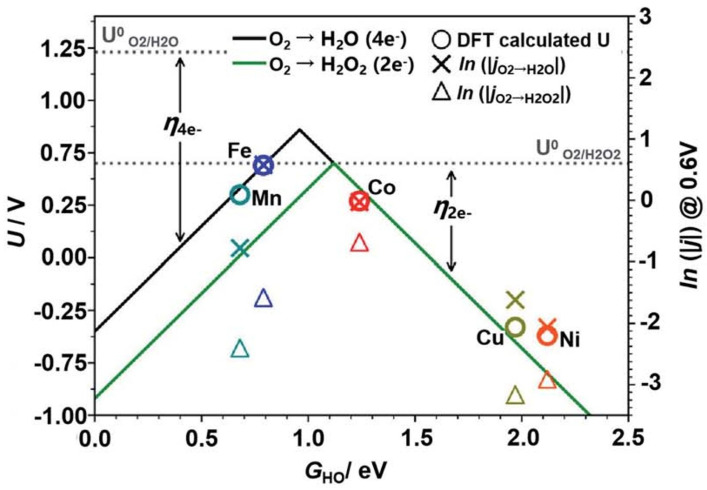
Volcano image of M-N-C catalysts. Reproduced with permission [63]. Copyright 2013, Royal Society of Chemistry.

Despite the distinct (Pt-like) electronic characteristics displayed by M-N-C catalysts, significant challenges remain in replacing Pt-based catalysts for large-scale ORR applications. The catalytic performance of M-N-C catalysts is mainly influenced by three key factors: (1) Thermal migration and aggregation of metal atoms lead to the formation of low-activity nanocrystalline materials. (2) The strong binding between oxygen-containing intermediate species and metal active sites leads to a decreased ORR reaction rate, as illustrated in Figure 4. M-N-C is primarily situated on the left region of the volcano plot, suggesting that the binding interaction between M-N-C and *OH is overly strong, thereby impeding desorption and ultimately influencing catalytic performance [63]. (3) The uneven arrangement of pore structures across the catalyst surface restricts the efficient transport of oxygen-containing intermediate species [64,65]. In addition, corrosion of the carbon framework caused by oxidation results in the leaching of metal active sites, thereby compromising catalytic stability [66]. Thus, additional refinement of the microstructural and electronic configurations of M-N-C catalysts is necessary to improve their catalytic performance and durability in ORR.

### 2.2. Strategies for Enhancing the Catalytic Activity of M-N-C Catalysts

Currently, researchers are primarily exploring two strategies to boost the catalytic efficiency of M-N-C catalysts: (1) enhancing the concentration of M-N_x_ coordination centers to expand the interaction area between catalytic sites and oxygen-containing intermediate species, thereby accelerating the ORR rate; (2) enhancing the intrinsic catalytic performance by tuning the electronic configuration and coordination surroundings of the metal center, thereby promoting oxygen adsorption and the conversion of reaction intermediates.

#### 2.2.1. Increasing the Density of M-N_x_ Sites

**Figure 5:**
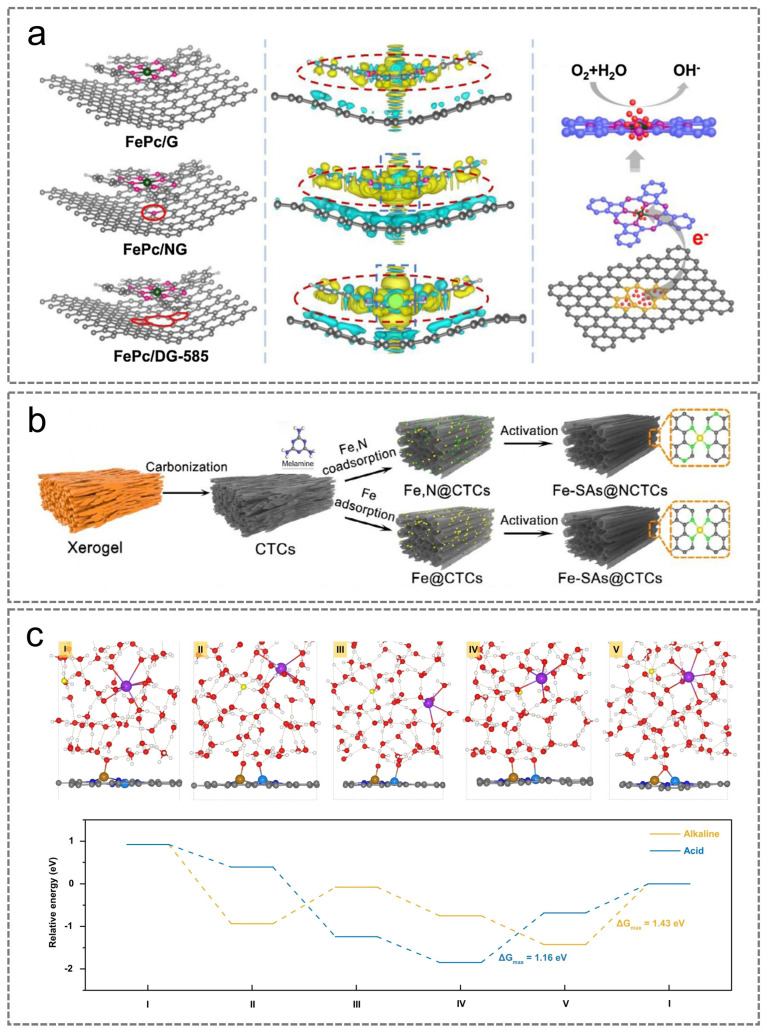
(**a**) The catalyst models and charge densities for FePc/G, FePc/NG, and FePc/G585. Reproduced with permission [74]. Copyright 2019, Elsevier. (**b**) Preparation illustrations of Fe-SAs@CTCs. (**c**) Typical structural configurations observed at the interface during the ORR process in alkaline environments. (**b**,**c**) Reproduced with permission [62]. Copyright 2023, Springer Nature.

Owing to the elevated surface energy of transition metal single atoms, they tend to cluster together and undergo oxidation throughout the synthesis and catalytic reaction stages, presenting a significant challenge in the preparation of highly atomically dispersed M-N-C catalysts [66,67]. Therefore, it is essential to restrict the free migration of M atoms to ensure their stabilization through M-N bonding within the carbon support [68]. It is worth noting that the strength of the M-N bond directly influences the loading capacity of the M atoms. Nitrogen-containing organic compounds can donate lone pairs of electrons to N atoms, which exhibit strong coordination affinity toward M atoms, thereby promoting the formation of stable M-N bonds. Porphyrins [69] and phthalocyanines [70] offer a well-defined non-coordinating environment that enables stable binding with M atoms, resulting in the generation of well-dispersed M-N-C active sites. In addition, 2D sheet-like nanographene with a unique structure is considered a promising candidate for hosting M-N_x_ sites owing to its large specific surface area, superior electrical conductivity, and unique graphite support [65,71]. Graphene inherently lacks active sites that are favorable for doping owing to its chemical inertness. Conversely, graphene with nitrogen doping or structural defects can promote robust π–π stacking interactions, thereby enabling enhanced coordination for anchoring metal atoms, which promotes the generation of isolated single-metal-atom catalytic centers [72,73]. Chen et al. constructed Fe/N-doped electrocatalysts by assembling nitrogen-doped graphene nanosheets with single iron atoms (Figure 5a) [74]. The Fe atoms anchored on the catalyst coordinate with nitrogen atoms in graphene to form stable Fe-N-NG structures. High-temperature pyrolysis and acid leaching generate single-atom dispersed Fe-N_x_ sites, while the short-range order and large interlayer spacing of the Fe-N-NG structure facilitate O_2_ diffusion, thereby contributing to enhanced ORR catalytic activity. In addition, Chen et al. successfully developed a high-performance and stable Mn-N-C oxygen reduction catalyst via a three-step synthetic approach integrating chemical doping, mechanochemical doping, and pyrolysis. This synthetic strategy not only significantly enhanced the doping content and density of manganese but also established a structural framework conducive to the synergistic enrichment of manganese and nitrogen species [75].

In addition to stabilizing individual metal atoms via strong interactions between M atoms and carbon supports, nitrogen-rich precursors with cross-linking capabilities can also suppress metal atom migration during high-temperature pyrolysis and facilitate their uniform distribution on the support surface, ultimately contributing to the formation of densely populated M-N-C active sites. MOFs composed of metal nodes and nitrogen-enriched ligands not only provide nitrogen-based coordination environments for metal atoms, but also yield carbon matrices with large surface areas and well-organized porous architectures following high-temperature calcination, which promotes the accessibility of more active centers [21,59,66,76,77]. Fe^3+^ ions were incorporated into a Zn-based MOF through chemical doping to form a metal complex, which was subsequently utilized for the preparation of an Fe-N-C catalyst featuring Fe-N_4_ active sites via pyrolysis [27]. The Fe doping content not only has a direct impact on the ORR activity, but also exerts an indirect influence on the catalytic performance by altering key factors including particle dimensions, pore architecture, and nitrogen concentration. Although the encapsulation of metal atoms through enhanced metal–substrate interactions or the construction of nanocage structures represents an effective strategy for stabilizing metal atoms, this approach depends on specific support materials and synthesis conditions, thus limiting its broad applicability to some extent.

Apart from the suppression effect induced by the template, combining multiple stabilization approaches synergistically can more effectively prevent the formation of metal clusters, thus enabling the realization of high metal-atom loading. The stepwise co-anchoring method introduced by Li and colleagues offers a practical solution for the scalable production of M-N-C catalysts featuring high metal-atom loading (Figure 5b,c) [62]. Initially, metal ions form complexes with chelating agents (such as melamine and dicyandiamide) and are immobilized on a high-surface-area porous carbon substrate, offering initial stabilization for the metal atoms. Following this, the surplus chelating agents present on the support surface create a physical barrier around the metal complexes, providing an additional layer of stabilization. The residual products formed during the thermal decomposition of the chelating agent-bound metal complexes help further disperse the metal atoms, acting as a third level of protective stabilization. Meanwhile, N-containing carbon frameworks are able to bond with metal atoms during the thermal decomposition process, resulting in the formation of M-N_x_ active sites, which effectively inhibit the clustering of metal atoms.

In recent years, M-N-C composites have garnered considerable interest owing to their exceptional atomic efficiency and superior catalytic capabilities. However, traditional synthesis methods face numerous challenges, including high energy consumption, complicated procedures, and significant waste of metal materials, all of which severely hinder their practical application [70,78,79]. Therefore, the scalable and versatile synthesis of highly efficient M-N-C catalysts represents a critical step toward reducing production costs and accelerating industrialization. Mechanochemistry is an effective synthesis technique that utilizes mechanical energy to induce chemical reactions among different solid materials. Ball milling is one of the key techniques for realizing mechanochemical synthesis. It involves repeatedly compressing materials using hard steel or oxide balls to trigger chemical reactions at the interfaces of reactants under relatively mild temperature conditions. Compared with other kilogram-scale methods for preparing single-atom catalysts (SACs), the mechanical ball milling approach for synthesizing M-N-C materials offers several notable advantages [80]: (1) Metal atoms can be evenly distributed across the support, attaining a single-atom dispersion state. (2) High-quality and high-density M-N-C sites with defective scaffolds can be prepared for multifunctional catalytic applications. (3) M-N-C materials can be synthesized without the need for solvents or post-synthesis heat treatment, offering enhanced environmental compatibility. (4) This approach is straightforward and effective, enabling the large-scale production of catalysts at the kilogram level without compromising their structural integrity.

#### 2.2.2. Enhancing Intrinsic Activity

Improving the inherent activity of the active centers is essential for further enhancing the catalytic efficiency of M-N-C materials. A typical M-N-C catalyst features a central metal atom coordinated by adjacent nitrogen atoms within the carbon matrix. Additionally, heteroatoms such as sulfur (S), oxygen (O), and phosphorus (P) can be incorporated into the carbon skeleton to create diverse coordination environments. In M-N-C catalysts, M atoms serve as active centers and interact directly with oxygen intermediates. First-order coordinating atoms bond to M atoms and participate in electron sharing, whereas second-order or higher-order coordinating atoms (collectively referred to as the coordination environment) can adjust the electronic configuration of M-N-C sites through extended electron delocalization. Additional functional groups can also affect the electronic environment of M-N-C active sites via coordination linkages or non-covalent intermolecular forces. Thus, the intrinsic activity of M-N-C sites for the oxygen reduction reaction can be substantially improved by carefully choosing the central metal atom and coordinating elements along with fine-tuning the coordination environment and incorporating suitable auxiliary groups.

**Figure 6:**
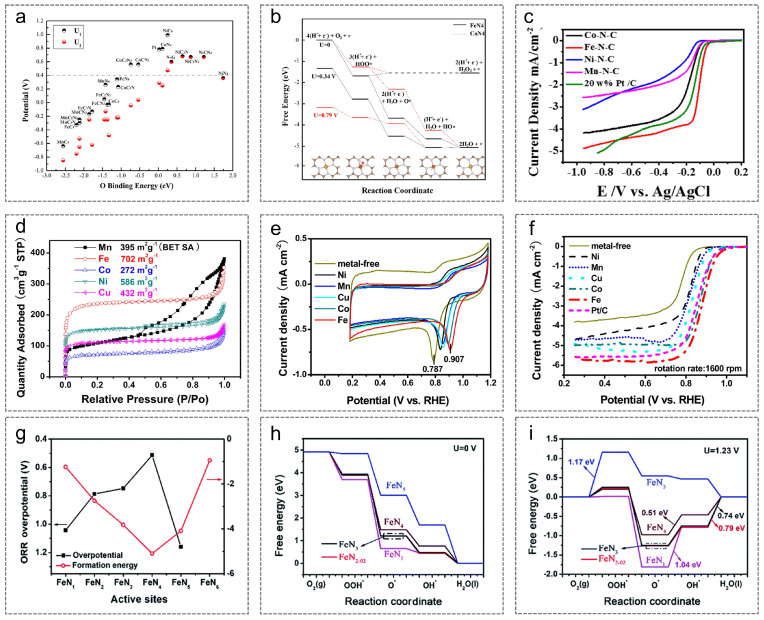
(**a**) U1 and U2 values on different square-planar active centers are plotted as a function of the oxygen binding energy; (**b**) DFT results; (**c**) LSV curves. (**a**–**c**) Reproduced with permission [81]. Copyright 2016, Elsevier. (**d**) Nitrogen adsorption–desorption curve; (**e**) CV curves and (**f**) LSV curves in 0.1 M KOH. (**d**–**f**) Reproduced with permission [82]. Copyright 2014, American Chemical Society. (**g**) Formation energies; ORR free energy curves of FeN*_x_* at (**h**) 0 V and (**i**) 1.23 V. (**g**–**i**) Reproduced with permission [93]. Copyright 2013, Royal Society of Chemistry.

① Choosing a suitable metal center:

The active sites in M-N-C catalysts are typically metal atoms embedded within the carbon matrix. The electronic structure of these metal atoms plays a crucial role in the ORR, as it determines the adsorption strength of oxygen-containing intermediates on the metal surface, thereby influencing the intrinsic catalytic activity. For transition metals, the filling state of their *d* orbitals, as well as the interaction between these *d* orbitals and the *p* orbitals of oxygen atoms in oxygen-containing intermediates, directly determines the adsorption behavior of the intermediates and the rate of electron transfer during the ORR process. Zheng and colleagues explored the oxygen reduction reaction (ORR) performance of graphene-supported M-N-C catalysts by integrating computational modeling with experimental analysis. Their findings revealed that Fe-N-C catalysts displayed the most superior ORR performance compared with Co-N-C, Mn-N-C, and Ni-N-C catalysts (Figure 6a–c) [81]. Peng and colleagues further studied the impact of Fe(acac)_3_, FeCl_3_, and Fe(NO_3_)_3_ on Fe-N-C catalyst performance (Figure 6d–f) [82]. Due to their stronger hydrolysis capabilities, Fe(acac)_3_ and FeCl_3_ metal salts enable the synthesis of highly dispersed FeN_x_ sites, whereas Fe(NO_3_)_3_, with weaker hydrolysis ability, leads to the formation of inactive Fe_3_C after thermal reduction. The results demonstrated that the catalysts synthesized from three distinct metal salts displayed varying catalytic performances with the following activity sequence: Fe(acac)_3_ > FeCl_3_ > Fe(NO_3_)_3_ [83]. Thus, Fe is considered the most favorable central metal in M-N-C catalysts for the oxygen reduction reaction, and Fe(acac)_3_ exhibits the best performance among metal salt precursors. Zhang et al. revealed that distinct metal active centers lead to diverse adsorption configurations of oxygen-containing intermediates, thereby exerting a significant influence on the ORR activity of M-N-C catalysts [84]. In the case of Ni-N-C catalysts with lower binding energy, *O preferentially adsorbs at the M-N bridge site, and this preferential adsorption plays a crucial role in enhancing the ORR activity. In addition, Ruan et al. reported a highly efficient binary FeV-DAS-NC catalyst, wherein the synergistic interaction between Fe and V metal atoms markedly enhances the ORR activity [85].

② Regulation of metal coordination atoms:

Crystal field theory suggests that coordination atoms interact with central metals to create coordination field effects, which adjust metal bonding energy, impact oxygen intermediate adsorption at metal sites, and thus affect ORR catalytic performance. Typically, the nitrogen coordination structures present at M-N-C active sites are mainly composed of pyridinic, pyrrolic, graphitic, and oxidized nitrogen forms. These distinct configurations demonstrate varying adsorption behaviors toward oxygen intermediates [86,87,88]. Among these configurations, pyridinic nitrogen not only coordinates with metals to form active sites for ORR, but also activates adjacent carbon atoms, converting them into additional active sites.; pyrrolic nitrogen enhances electron transfer around metal atoms, thereby balancing the adsorption and desorption behaviors of oxygen intermediates [89]; graphitic nitrogen adjusts the electrical conductivity of the catalyst and facilitates the mass transfer of reaction products, thereby favoring the four-electron ORR mechanism and enhancing the catalyst’s intrinsic ORR activity [90]. Zhang et al. successfully designed and synthesized a novel Co/Cu-N-C catalyst via a complex-assisted pyrolysis strategy [91]. The precise modulation of the coordination environment around the Co sites by the Cu-N carrier resulted in a significant enhancement of catalytic activity.

In addition to the influence of the N configuration, the number of nitrogen atoms also plays a role in modulating the charge distribution of metal sites, thereby affecting the adsorption strength between metal active sites and oxygen-containing species [92]. For example, theoretical calculations were employed to predict the optimal adsorption configuration at Fe-N_x_ sites (x = 1–5) (Figure 6g–i) [93]. Among all Fe-N_x_ structures, the Fe-N_4_ configuration exhibits the lowest adsorption barrier for the key oxygen-containing intermediate (i.e., *OH). With the coordination number rising from 1 to 4, the ORR overpotential gradually diminishes. However, when the nitrogen coordination number further increases to 5, the ORR overpotential actually rises. Therefore, the ORR activity and nitrogen coordination number in Fe-N_x_ catalysts exhibit a “volcanic” relationship.

**Figure 7:**
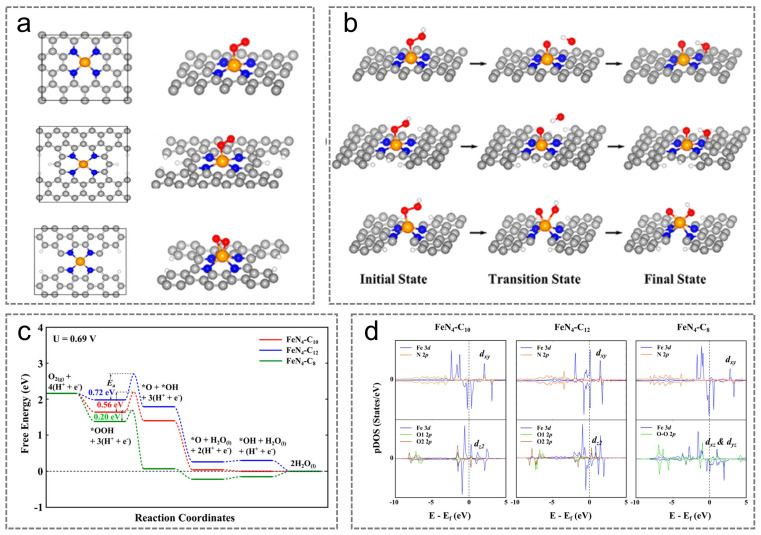
(**a**) Atomic structures and O_2_ adsorption at the ORR active sites of Fe-N_4_-C_10_ (D1), Fe-N_4_-C_12_ (D2), and Fe-N_4_-C_8_ (D3); (**b**) atomic structures of the *OOH decomposition reaction’s initial, transition, and final states on D1, D2, and D3; (**c**) optimized free energy profiles for ORR; (**d**) PDOS for different catalysts. (**a**–**d**) Reproduced with permission [95]. Copyright 2017, American Chemical Society.

③ Regulation of the remote coordination environment:

M-N-C catalysts enable remote atoms and functional groups within the carbon matrix to interact via long-range electron delocalization effects. Therefore, modulating the electronic properties of the carbon matrix can influence the electronic structure of the metal center, thereby enhancing the intrinsic activity of the M-N-C catalyst. The formation of M-N-C catalysts is primarily determined by metal precursors, organic carbon sources, and pyrolysis conditions. Pyrolysis serves as a critical process for constructing the complex architecture of M-N-C materials [94,95,96]. Liu et al. identified three unique FeN_4_ active sites, each exhibiting different local carbon structural coordination and formed at varying pyrolysis temperatures, denoted as Fe-N_4_-C_10_ (D1), Fe-N_4_-C_12_ (D2), and Fe-N_4_-C_8_ (D3) (Figure 7a) [95]. Generally, at low temperatures, D2 is the predominant active site in Fe-N-C catalysts. As the temperature increases, the relative abundance of D1 rises compared with D2. Upon heating to a certain temperature, D1 dissociates from D2 and is subsequently recaptured at vacancy defects or edges, leading to the formation of new D1 sites. Electrochemical results demonstrate that D1 exhibits higher ORR activity compared with D2. This phenomenon can be attributed to the Fe^2+^/Fe^3+^ redox pair formed during high-temperature pyrolysis, which possesses a high redox potential and thereby contributes to the stability of the catalytically active Fe^2+^ under elevated potential conditions. D3 originates from microporous carbon materials and displays behavior opposite to D1, exhibiting higher intrinsic activity compared with both D1 and D2 [95]. Meanwhile, D3, characterized by abundant edge microporous structures, helps further boost the intrinsic ORR activity of the Fe-N-C catalysts. Based on this analysis, studies of the catalytic mechanisms of D1, D2, and D3 (Figure 7b) reveal that D3 located at micropore edges exhibits the lowest O-O bond cleavage activation energy (0.20 eV), considerably smaller compared with D1 (0.50 eV) and D2 (0.80 eV). Moreover, D3 follows an ideal four-electron reaction pathway.

Analysis of the catalytic reaction behavior of D1, D2, and D3 reveals that the *OOH dissociation process on D1 and D2 involves the movement of the *OH product to the apex of a neighboring carbon atom. Conversely, the edge microporous architecture of D3 promotes the breaking of the O-O bond and the dissociation of *OOH at the metal center. This behavior causes D3 to have a smaller reaction barrier for converting *OOH to *O and *OH (Figure 7c). Based on these findings, Chen et al. further explored the influence of the chemical properties of adjacent nitrogen or carbon atoms surrounding M-N_x_ sites on the catalytic activity of M-N-C catalysts. The results demonstrate that, within the same D2 configuration, the ORR activity of active sites formed under NH_3_ pyrolysis conditions is significantly higher than that of active sites generated under Ar pyrolysis conditions. The reason for this difference lies in the fact that pyrolysis under NH_3_ atmosphere enhances the formation of nitrogen species with high alkalinity on the carbon support (Figure 7d), which is associated with the high degree of electron delocalization on the carbon-based surface and its strong electron-donating capability. This enhances the adsorption between the central metal atom and oxygen-containing ORR intermediate, boosting D2’s intrinsic ORR activity. Based on the aforementioned research findings, alterations in the coordination environment of the carbon skeleton within M-N-C catalysts can modulate their dissociation behavior toward reaction intermediates, thereby improving their intrinsic catalytic activity [94]. Therefore, the synergistic effects of selecting optimal metal active centers and optimizing their coordination environment (including the type and number of first-shell coordination sites and remote regulation by carbon supports) can significantly improve the catalytic performance of M-N-C catalysts.

In M-N-C catalysts, the simultaneous achievement of high active-site density and high intrinsic activity may involve addressing the following key challenges: (1) preventing metal atoms from aggregating under high metal-loading conditions while maintaining their atomic dispersion state; (2) precisely controlling the coordination environment to ensure uniform electronic structures across all active sites; (3) achieving a balance between constructing a porous structure favorable for mass transfer and maintaining adequate electrical conductivity.

### 2.3. Strategies for Enhancing the Stability of M-N-C Catalysts

**Figure 8:**
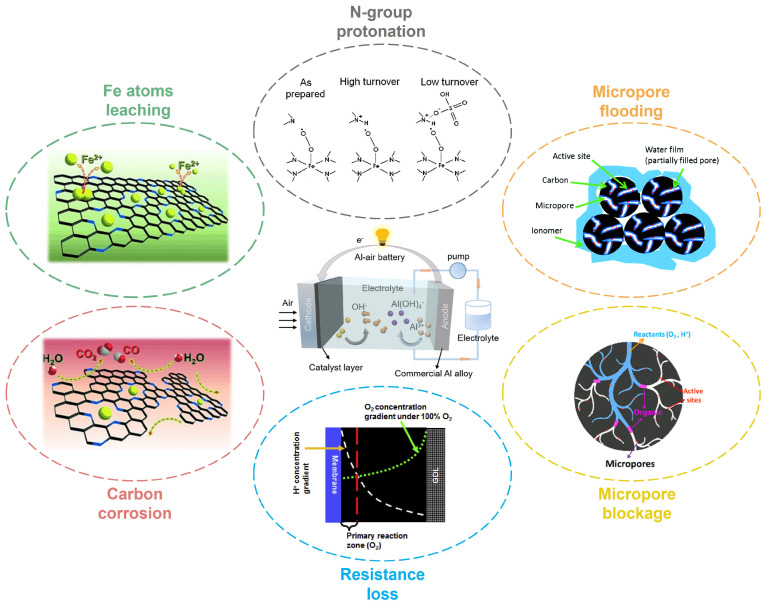
Factors contributing to the degradation of Fe-N-C catalyst performance. Images for “Fe atoms leaching” and “Carbon corrosion”: Reproduced with permission [101]. Copyright 2015, Wiley-VCH. Images for “N-group protonation”: Reproduced with permission [102]. Copyright 2011, American Chemical Society. Images for “Micropore flooding”: Reproduced with permission [103]. Copyright 2017, Royal Society of Chemistry. Images for “Micropore blockage”: Reproduced with permission [104]. Copyright 2018, American Chemical Society. Images for “Resistance loss”: Reproduced with permission [105]. Copyright 2017, Elsevier. Images for “Al–air battery”: Reproduced with permission [106]. Copyright 2024, American Chemical Society.

In practical applications, the stability of M-N-C catalysts remains an area with substantial room for improvement. Studies show that Fe-N-C catalysts demonstrate superior catalytic performance compared with other transition metal M-N-C catalysts in alkaline ORR [26,97,98,99]. Therefore, enhancing the stability of Fe-N-C catalysts plays a critical role in the development of high-performance M-N-C catalysts suitable for industrial applications. The catalytic performance of Fe-N-C catalysts is challenging to maintain efficient, and the associated specific activity decay can be categorized into two distinct stages: an initial rapid decline in catalytic activity followed through a gradual decline over extended electrocatalytic processes [100]. To preserve the catalytic performance of the catalyst, it is essential to prevent or mitigate the aforementioned degradation process. As illustrated in Figure 8, the primary factors contributing to the decline in stability can be summarized as follows: (1) micropore flooding, (2) Fe leaching, (3) N-group protonation, (4) carbon corrosion, (5) resistance loss, and (6) micropore blockage [50]. To date, strategies to enhance catalyst stability have been advanced through two primary approaches: enhancing the electronic characteristics of Fe-N-C active centers while strengthening the oxidative resistance of the carbon support.

#### 2.3.1. Enhancing Electronic Effect

**Figure 9:**
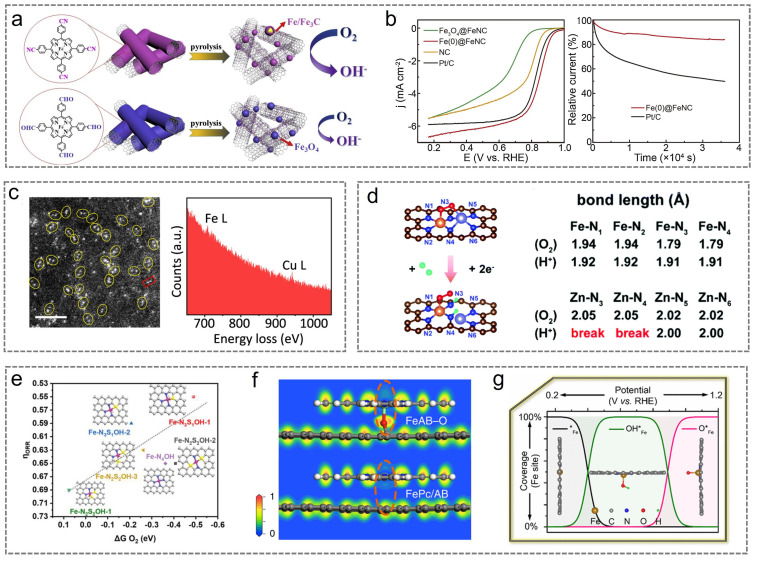
(**a**) Schematic illustrations of Fe(0)@FeNC; (**b**) catalyst activity and stability of Fe(0)@FeNC. (**a**,**b**) Reproduced with permission [108]. Copyright 2016, American Chemical Society. (**c**) TEM and EELS images of Fe/Cu-N-C. Reproduced with permission [109]. Copyright 2023, American Chemical Society. (**d**) ORR process of Fe/Zn-N-C. Reproduced with permission [111]. Copyright 2008, Royal Society of Chemistry. (**e**) Calculated free energy evolution diagrams for S-Fe-N_4_. Reproduced with permission [112]. Copyright 2022, Wiley-VCH. (**f**) ELF images of FeAB-O. Reproduced with permission [113]. Copyright 2020, Springer Nature. (**g**) ORR process of OH-Fe-N_4_ catalyst. Reproduced with permission [114]. Copyright 2019, American Chemical Society.

The deactivation of M-N-C catalysts is primarily attributed to the dissolution or agglomeration of metal atoms. Enhancing the electronic interaction between the carbon substrate and metal sites facilitates the formation of a more stable coordination structure, thereby effectively suppressing metal atom dissolution and agglomeration. Additionally, modulating the electronic interaction between the carbon substrate and metal sites can optimize the electronic structure of metal atoms, thereby preventing active site poisoning caused by excessive adsorption of oxygen-containing intermediates. Some metal nanocrystals on the catalyst surface (such as metal atoms, metal oxides, metal nitrides, and metal carbides) do not directly function as active sites; instead, they influence the charge transfer capability of Fe-N_x_ sites, thereby enhancing catalytic performance. These nanoparticles serve as supplementary elements that can influence the electronic configuration of Fe-N-C catalysts. Fe/Fe_3_C nanocrystals alone do not confer high catalytic activity; however, catalysts incorporating both Fe/Fe_3_C nanocrystals and Fe-N_4_ sites demonstrate superior activity and stability [107]. After the Fe/Fe_3_C nanocrystals are removed, the catalytic activity and stability exhibit significant degradation, indicating that Fe/Fe_3_C nanocrystals contribute to enhancing the ORR performance of Fe-N_4_ sites (Figure 9a,b). Additionally, the interaction between Fe/Fe_3_C and Fe-N_4_ sites enhances oxygen adsorption and mitigates the detachment of Fe atoms during the complex ORR process [108].

The Fe atoms in the diatomic catalyst interact with other transition metal atoms, thereby enhancing the structural stability of the catalyst and mitigating Fe leaching during the reaction process. Additionally, diatomic catalysts can reduce the catalyst’s surface energy and improve its catalytic efficiency by adjusting atomic-level interactions. Zhang et al. synthesized ten bimetallic catalysts using macrocyclic precursors comprising six homonuclear species (Fe_2_, Co_2_, Ni_2_, Cu_2_, Mn_2_, and Pd_2_) and four heteronuclear counterparts (Fe-Cu, Fe-Ni, Cu-Mn, and Cu-Co). The Fe-Cu bimetallic catalyst demonstrated exceptional stability, attributed to its suppression of the Fenton process (with only a 12 mV E_1/2_ loss after 30,000 cycles, Figure 9c) [109]. Qiu et al. investigated charge transfer between two heteronuclear metal atoms using density functional theory (DFT) [110]. By comparing the number of electrons transferred across different *d* orbitals, they successfully identified the CoFe-N-C bimetallic atomic site catalyst with optimal performance and elucidated the cooperative electronic interaction between Fe and Co during the ORR catalytic process. Charge transfer readily occurs between adjacent metal atoms, efficiently adjusting the electronic configuration of the metallic active site Fe, optimizing the adsorption energy of reaction intermediates, and thereby significantly enhancing ORR performance. Li et al. proposed a unique semi-metallic electronic structure based on theoretical screening and rational design of Fe/Zn-N-C double-atom catalysts (Figure 9d) [111]. This structure, formed through the synergistic interaction of Fe and Zn, introduces spontaneous spin polarization at the Fermi level, thereby enhancing electron conductivity and facilitating the capture and bonding of free oxygen. Meanwhile, during the ORR process, the FeN_4_ active center remains intact as a result of the sacrificial ZnN_4_ site. The 2*H model of the FeZnN_6_ site in the Fe/Zn-N-C diatomic catalyst confirmed that the N3 and N4 positions selectively coordinate with protons, resulting in the cleavage of the Zn-N bond. After protonation, slight increases in the bond lengths of Fe-N3 and Fe-N4 were observed (from 1.79 Å to 1.91 Å), which may help preserve the FeN_4_ site from degradation, thereby supporting a continuous and sustainable ORR process. Additionally, the protonation step leaves the electronic configuration of the FeN4 reaction site unchanged (but instead converts ZnN_4_ to ZnN_2_), thereby preserving the fully spin-polarized charge carriers present at the Fermi energy and maintaining its catalytic reactivity.

Introducing axial ligands into the metal center to modulate the electronic structure of the catalytic site effectively suppresses the detachment of metal sites. Typically, the *d* orbitals associated with the central metal atoms that are perpendicular to the Fe-N-C plane remain partially unoccupied and are capable of interacting with external ligands. In Fe-N-C catalytic systems, axial coordination between metal atoms and ligands including P, O, S, and OH groups can occur. These ligands are capable of altering the electronic configuration of metal centers, which in turn helps improve the stability of the catalyst. For example, in the S-Fe-N_4_ catalyst, a single Fe atom is coordinated with four pyridinic nitrogen atoms and forms an axial bond with sulfur. Axial coordination with sulfur alters the electronic configuration of the iron center, leading to improved structural stability of the catalytically active regions (Figure 9e) [112]. Moreover, axially coordinated oxygen atoms can break the electronic symmetry of iron, leading to localized electron accumulation and thus facilitating O_2_ adsorption as well as enhancing charge transfer between the active centers and oxygen-containing molecules (Figure 9f) [113]. The O-Fe-N4 catalyst featuring axial oxygen coordination exhibits remarkably enhanced catalytic performance and remarkable stability, significantly outperforming commercial Pt/C.

Apart from external ligands, ORR intermediates can also naturally coordinate with the central metal atoms, generating internal auxiliary groups. By modulating the planar or axial coordination of oxygen intermediates at the central metal site, the ORR performance can be significantly enhanced. Within the potential range of 0.28 to 1.00 V, Fe atoms spontaneously coordinate with *OH intermediates. The OH-Fe-N_4_ structure formed by the dynamic coverage of *OH intermediates acts as the true ORR active site, demonstrating superior performance compared with the original Fe-N_4_ site (Figure 9g) [114]. *OH can modulate the electronic configuration of Fe and modulate the binding and release of reaction intermediates. However, this electrochemical in situ condition strategy is not without limitations, as *OH axial coordination enhances the selectivity toward H_2_O_2_, thereby compromising the stability of the catalyst. Han et al. synthesized Cl-Fe-N_4_-CS catalysts that exhibited enhanced ORR catalytic performance [115]. The Cl-Fe-N_4_-CS catalyst exhibited an E_1/2_ value of 0.921 V, with no degradation in E_1/2_ observed after 10,000 cycles of CV testing, significantly surpassing both the E_1/2_ of Fe-N_4_-C and the performance of commercial Pt/C. Density of states analysis indicates that axial coordination of Cl and S doping in the carbon skeleton enhances hybridization between the Fe 3*d* orbitals at the active center and the O 2*p* orbitals of the catalyst, which facilitates acceleration of the ORR. Among them, Cl coordination predominantly contributes to high catalytic activity, whereas S doping primarily accounts for the enhanced stability of Fe-N-C catalysts. In summary, axial coordination involving metal atoms represents a viable strategy for enhancing the stability of Fe-N-C catalysts.

#### 2.3.2. Enhancing the Antioxidant Capacity of Carbon-Based Materials

**Figure 10:**
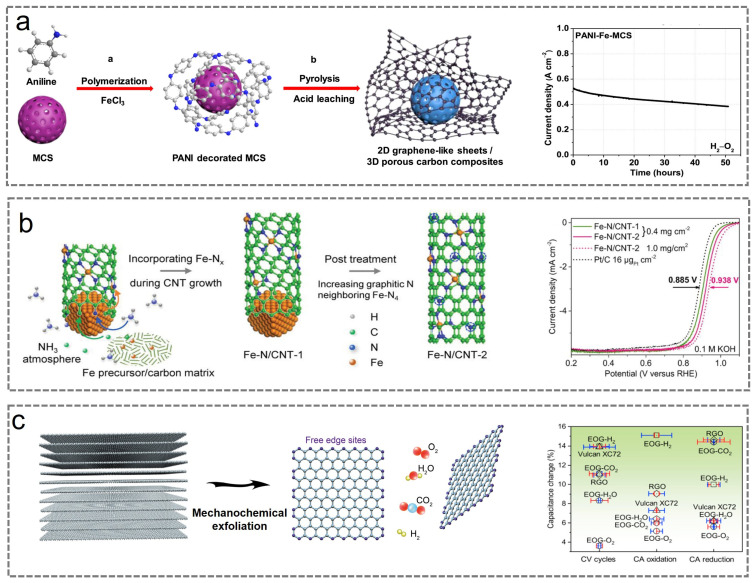
(**a**) Schematic illustrations and stability of PANI-Fe-MCS. Reproduced with permission [122]. Copyright 2017, Elsevier. (**b**) Schematic illustrations and stability. Reproduced with permission [123]. Copyright 2019, Wiley-VCH. (**c**) Schematic illustrations and stability of EOG-O_2_, EOG-H_2_O, EOG-CO_2_, EOG-H_2_. Reproduced with permission [124]. Copyright 2019, Elsevier.

Carbon substrate corrosion represents one of the primary factors contributing to the degradation of carbon-based catalyst performance, with its prevalence predominantly attributed to oxidative processes. Given the detrimental impact of carbon substrate corrosion on the stability of Fe-N-C catalysts, mitigating carbon substrate oxidation is essential. The primary strategies for enhancing the oxidation resistance of carbon substrates include carbon graphitization and edge functionalization. To date, the majority of Fe-N-C catalysts exhibit insufficient graphitization of the carbon matrix, leading to poor corrosion resistance and consequently resulting in active site degradation and a decline in turnover frequency (TOF) [116,117]. Additionally, carbon substrates with low graphitization exhibit poor electron transfer and conductivity [118]. Generally, porous carbon carriers exhibit a high specific surface area, yet their electrochemical oxidation resistance remains limited [119]. Graphitized carbon skeletons exhibit high resistance to electrochemical oxidation under high electrode potentials. They also effectively suppress the oxidation and waterlogging of catalytic active sites, thereby minimizing resistance losses during mass transfer. However, metal atoms tend to be poorly dispersed within these structures [120,121]. Therefore, the development of Fe-N-C catalysts featuring porous graphitic carbon frameworks enriched with Fe-N_x_ active sites represents a key challenge in achieving efficient and stable ORR. As shown in Figure 10a, Chen et al. developed a Fe-N-C catalyst (PANI-Fe-MCS) composed of thin carbon sheets and porous carbon spheres, exhibiting excellent catalytic performance [122]. A larger electrochemical active area, a reasonable pore size distribution, and a controllable chemical structure contribute to enhanced catalytic performance. PANI-Fe-MCS was employed as a cathode catalyst in an aluminum–air battery and subjected to long-term stability testing, thereby demonstrating its high efficiency and stability. As shown in Figure 10b, Kang et al. reported that carbon nanotubes incorporated dense Fe-N_x_ centers and were encapsulated by graphitic nitrogen, demonstrating high catalytic activity and stability [123]. The adjacent graphitic nitrogen facilitates improved *d*-band filling, thereby reducing the partial magnetic moment (spin density), while the incorporation of iron atoms preserves high catalytic activity. Meanwhile, mesoporous/macroporous carbon nanotubes are effective in suppressing water flooding behavior. Compared with the Fe-N-C catalyst derived from ZIF-8 with low graphitization, the carbon nanocomposite Fe-N/CNT-2 demonstrates reduced performance degradation.

Carbon materials typically exhibit edge defects, which are broadly classified into armchair and zigzag configurations. Edge sites generally display different thermodynamic and electrochemical characteristics compared with the basal plane [125,126]. Generally, carbon oxidation predominantly occurs at defect-rich edges. Electrocatalysts with exposed inner surfaces exhibit greater resistance to oxidation compared with those with exposed edge structures [126]. Therefore, controlling the edge structures of the carbon substrate represents an effective strategy for mitigating carbon corrosion. Baek et al. employed O_2_, H_2_O, CO_2_, and H_2_ to selectively trim the free edges of graphene nanosheets through in situ or ex situ approaches, resulting in catalysts functionalized with edge oxidation groups at distinct positions, designated as EOG-O_2_, EOG-H_2_O, EOG-CO_2_, and EOG-H_2_ (Figure 10c) [124]. Accelerated chemical corrosion tests and electrochemical stability assessments demonstrated that the EOG-O_2_ catalyst featuring an ether ring structure ((G)C-OC-C(G)) exhibited superior electrochemical and chemical oxidation stability. Samples containing abundant hydrogen-containing functional groups are prone to further oxidation and exhibit inferior electrochemical stability. Among these groups, the (G) C–COOH functional group is highly susceptible to oxidative destruction, thereby exhibiting the lowest energy barrier. Therefore, current reports indicate that controlling the distribution of defects represents an effective strategy for preventing corrosion of the carbon matrix, thereby enhancing its electrochemical and chemical stability [49,127].

## 3. Summary and Outlook

Despite being regarded as some of the alkaline ORR catalysts with the most potential, M-N-C materials still fall short in terms of catalytic activity and stability for widespread commercial use. This paper provides a systematic overview of the general strategies employed in recent years to enhance the catalytic activity and stability of M-N-C catalysts. In terms of enhancing catalytic activity, the primary strategies include increasing the number of active sites in the catalyst (i.e., enhancing the M-N_x_ site density) and improving the intrinsic activity of these active sites, such as by selecting appropriate metal centers, adjusting the coordination atoms around the metal, and optimizing the remote coordination environment. The crucial factor in developing high-efficiency M-N-C catalysts lies in maximizing the number of active sites while maintaining their highest possible intrinsic activity. Additionally, the stability of M-N-C catalysts represents another critical factor influencing their commercialization potential. Currently, strategies to enhance the stability of M-N-C catalysts primarily focus on strengthening the electronic interactions between the carbon substrate and active sites as well as improving the antioxidant properties of the carbon substrate. In theory, further enhancement of the catalytic activity of M-N-C catalysts is achievable alongside improvements in their stability. This could also represent one of the key design principles for future M-N-C catalysts. Additionally, to accelerate the large-scale commercialization of M-N-C catalysts, further research efforts are likely needed in the following areas in the coming phase:

(1) Performance testing of M-N-C catalysts under actual operating conditions deserves greater emphasis. At present, the testing conditions for most M-N-C catalysts deviate substantially from their real-world operating environments. Increased focus on evaluating catalyst performance under actual operating conditions can significantly expedite their commercialization. To ensure the reliability of M-N-C catalyst performance during the transition from laboratory research to practical applications, the following key parameters should be prioritized in evaluation to bridge the gap between experimental conditions and real-world operating environments: (a) catalytic stability under high current densities; (b) mechanical stability of the catalyst structure; (c) the catalyst’s adaptability to dynamic operating conditions.
(2) Achieving mass production of M-N-C catalytic materials remains a key challenge. Most of the literature reports on the synthesis of M-N-C catalysts are still limited to the laboratory scale. Scaling up M-N-C catalysts from laboratory-scale synthesis to industrial applications presents the following technical challenges: (a) Dispersibility and agglomeration of metal nanoparticles. Under laboratory conditions, uniform dispersion of single metal atoms can be achieved by precisely controlling the precursor ratio and reaction parameters. However, during the scaling-up process, the uniformity of the precursor mixture decreases, which can easily cause metal atom agglomeration and reduce the density of active sites. (b) Controlled synthesis of porous structures. The performance of M-N-C catalysts relies on a high specific surface area and well-defined hierarchical pore structures (micropores, mesopores, macropores). However, during large-scale synthesis, the uniformity and reproducibility of these pore structures become challenging to maintain, which compromises mass transfer efficiency and limits the exposure of active sites. (c) Temperature gradient challenges in industrial high-temperature heat treatment processes. These thermal gradients can result in localized over-burning or insufficient carbonization, which disrupts the metal–nitrogen coordination structure and consequently diminishes catalytic activity. The template method effectively alleviates the problems faced by M-N-C catalysts in industrialization, such as poor metal dispersion, uncontrollable pore structure, and high cost, through structural pre-design, confinement effects, and in-situ doping strategies.
(3) Research on the in situ characterization of M-N-C catalysts under operational conditions should be prioritized. Techniques such as in situ Raman spectroscopy and in situ infrared spectroscopy are capable of directly elucidating the reaction pathways of M-N-C catalysts during the ORR, facilitating the rapid identification of key kinetic-determining steps and thereby accelerating the resolution of technical bottlenecks. In situ Raman and infrared spectroscopies enable real-time monitoring of oxygen-containing intermediates adsorbed on the surface of M-N-C catalysts during the ORR. Analyzing variations in the adsorption concentration of oxygen-containing intermediates on the catalyst surface provides insights into identifying the rate-determining steps during the ORR.

## Figures and Tables

**Table 1:** A systematic comparative analysis of the performance of ORR catalysts documented in the recent literature in 0.1 M KOH.

Catalyst	E_1/2_ (V vs. RHE)	Tafel Slope (mV dec^−1^)	Stability (ΔE_1/2_ After CV Cycling)	Reference
Fe-N-C_NH4I_	0.92	52	12 mV@20K cycles	[20]
Fe_SA_-N-C	0.89	--	6 mV@5K cycles	[21]
FeN_x_/NC-S	0.92	61	--	[22]
FeN_4_-O-NCR	0.94	54	5 mV@5K cycles	[23]
FeNSC-2Fe	0.91	59	7 mV@8K cycles	[24]
FeN_4_-Te*n*	0.87	86	--	[25]
Fe-ISAs/CN	0.90	52	2 mV@5K cycles	[26]
SA-Fe-NHPC	0.90	72	1 mV@10K cycles	[27]
Fe-N/C-SAC	0.91	52	7 mV@5K cycles	[28]
Fe_SA_/B, N-CNT	0.93	62	--	[29]
Fe-SAs/N-C	0.90	56	2 mV@10K cycles	[30]
FeN_4_Cl_1_/NC	0.91	36	2 mV@5K cycles	[31]
MS-Co_SA_-NC	0.86	89	14 mV@10K cycles	[32]
Zr-N/O-C	0.91	71	5 mV@10K cycles	[33]
S-Cu-ISA/SNC	0.92	50	2 mV@5K cycles	[34]
CoN_4_/NG	0.87	70	--	[35]
Mn-N-C-OAc	0.94	64	11 mV@5K cycles	[36]
O-Zr-N-C	0.91	66	2 mV@40K cycles	[37]
Co_1_-N_3_PS/HC	0.92	31	1 mV@10K cycles	[38]
MnSAs/S-NC	0.92	62	1 mV@5K cycles	[39]
Cu-SA/SNC	0.89	--	1 mV@10K cycles	[40]
Mn SAs-N_4_	0.90	69	0 mV@8K cycles	[41]
Mn-SAS/CN	0.91	69	0 mV@5K cycles	[42]
Zn-SAs/UNCNs	0.91	38	6 mV@5K cycles	[43]
Ce SAs/PSNC	0.90	47	4 mV@5K cycles	[44]
Cu-Se DAs	0.91	31	16 mV@10K cycles	[45]

## Data Availability

Data are contained within the article.
